# Dual Indocyanine Green (ICG)-Soaked Coil Placement for the Precise Localization of a Non-subpleural Peripheral Lung Nodule: A Modified Technique in a Case Report

**DOI:** 10.7759/cureus.91116

**Published:** 2025-08-27

**Authors:** Sammy Onyancha, Ramin Lonnes, Peter Hollaus, Gernot Rohde, Waldemar Schreiner

**Affiliations:** 1 Pulmonology, St. Elisabethen Krankenhaus, Frankfurt, DEU; 2 Thoracic Surgery, St. Elisabethen Krankenhaus, Frankfurt, DEU; 3 Pulmonology, Universitätsklinikum Marburg, Marburg, DEU

**Keywords:** fluorescence, icg, indocyanine green, lung sparring sublobar resection, vats

## Abstract

Accurate localization of small pulmonary nodules is essential for successful minimally invasive resection. Conventional preoperative localization techniques, such as placement of indocyanine green (ICG)-soaked coils, are optimized for nodules in close proximity to the pleura. We report on a novel dual-coil approach in a 45-year-old non-smoking patient with a PET-avid lesion, incidentally discovered during a trauma workup. The 9 mm right upper lobe lesion was located peripherally but not subpleural, with partial endobronchial and central orientation. Using flexible bronchoscopy, two ICG-soaked embolization coils were deployed: one distally within the segment to mark the boundary, and one proximally, just adjacent to the lesion. This approach enabled the three-dimensional bracketing of the nodule, allowing for precise anatomical resection via uniportal video-assisted thoracoscopic surgery (VATS) without the need for thoracotomy. This technique shows promise in expanding the utility of fluorescence-guided thoracoscopic resection by allowing localization of small, centrally oriented peripheral lung nodules that are not amenable to traditional single-coil marking.

## Introduction

Surgical resection of small, non-palpable pulmonary nodules increasingly relies on preoperative localization techniques to guide thoracoscopic procedures. Without accurate intraoperative identification, surgeons may be forced to enlarge resections or convert to open thoracotomy, both of which can compromise parenchymal preservation and recovery. Reported conversion rates attributable to the failure of nodule identification during video-assisted thoracoscopic surgery (VATS) vary across series but range from 6% to 7% in contemporary cohorts [1]; historical reports show substantially higher conversion rates when nodules cannot be localized [2,3].

Multiple localization strategies are available, each with trade-offs. CT-guided hookwire and microcoil techniques achieve high technical success but differ in complication profiles. Meta-analytic and comparative data indicate that hookwire localization carries pneumothorax rates of around one-third of cases (35%), with additional risks of hemorrhage and wire dislodgement, whereas microcoils achieve similar or higher success rates with fewer complications and lower failure/dislodgement rates. Recent series also suggest higher overall success rates with microcoils (99%) compared to hookwires (93%) [3-5].

Bronchoscopic approaches avoid pleural punctures, thus reducing the pneumothorax rate. Electromagnetic navigation bronchoscopy (ENB)-guided dye marking has been shown to yield localization success rates of up to 95% with very low serious complication rates in systematic reviews [6,7].

Indocyanine green (ICG) fluorescence-guided surgery has gained popularity due to its safety profile [8-10], high visibility under near-infrared imaging, and compatibility with preoperative bronchoscopic coil placement [11,12].

Conventionally, a single ICG-soaked coil is bronchoscopically deployed near the pleural surface to assist in the localization of subpleural nodules during VATS. However, the detectability of ICG declines with increasing tissue depth. Most clinical systems effectively visualize signals within a range of roughly 5-10 mm (up to ~15 mm in some settings), while deeper targets experience marked attenuation. Experimental spectroscopy platforms can extend this range, but they are not routine in clinical practice. Consequently, single pleural-surface marks or fiducials perform well for subpleural targets yet are less dependable for deeper, non-subpleural lesions [13-16]. In such cases, failure to localize the lesion may lead to either more extensive parenchymal resection or even conversion to open thoracotomy.

Precise localization using ICG allows surgeons to perform lung-sparing sublobar resections, preserving healthy tissue without compromising oncologic outcomes. Furthermore, when integrated with minimally invasive surgical approaches such as uniportal VATS, this strategy reduces patient trauma, shortens hospital stays, and expedites recovery.

We present a case of a modified dual-coil approach in a young patient with a PET-avid upper lobe lesion that was radiologically peripheral but not subpleural. The technique enabled precise, targeted resection via uniportal VATS, avoiding thoracotomy and preserving uninvolved lung parenchyma.

## Case presentation

**Figure 1:**
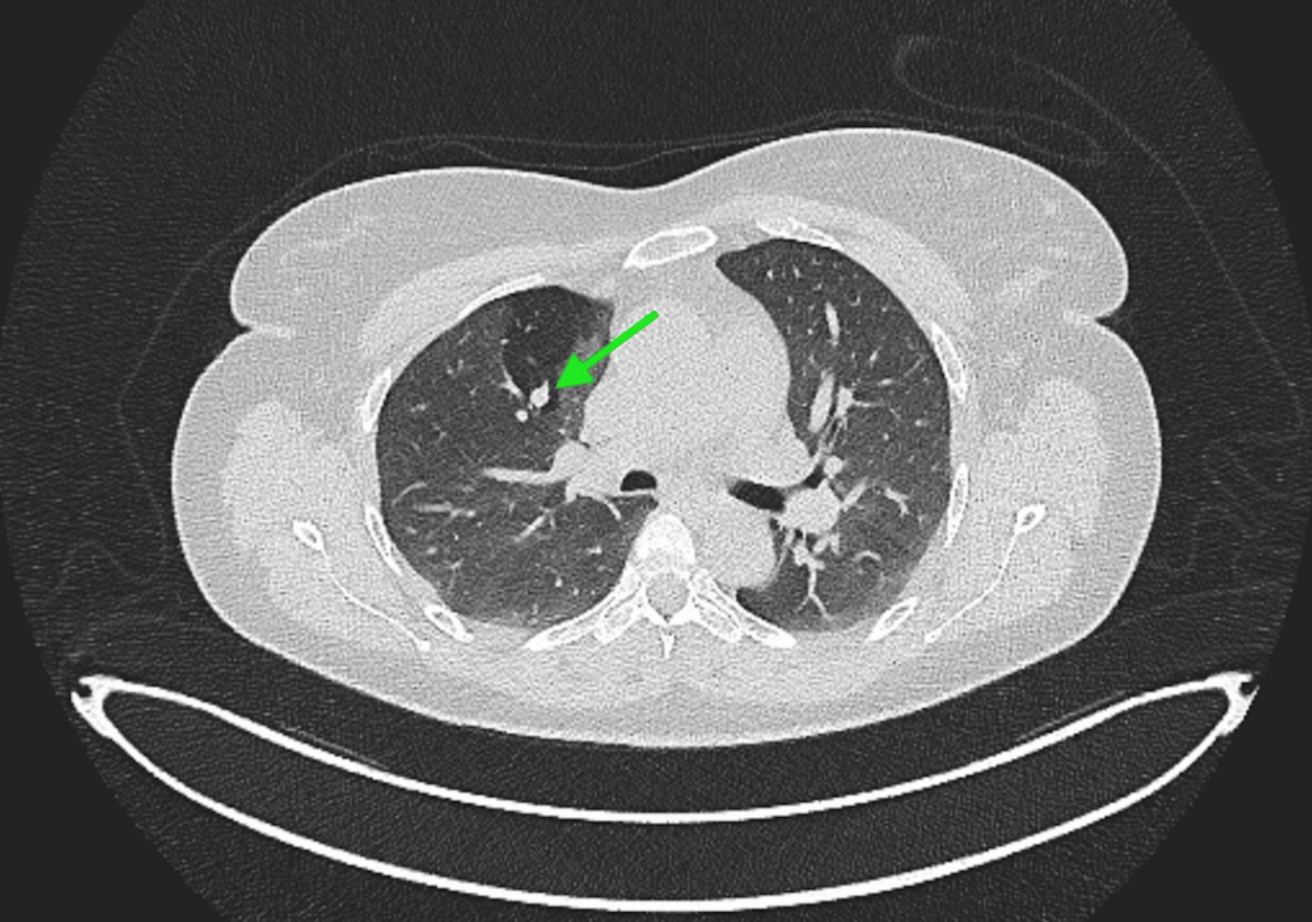
CT scan showing suspicion of lung cancer in the right lung.

**Figure 2:**
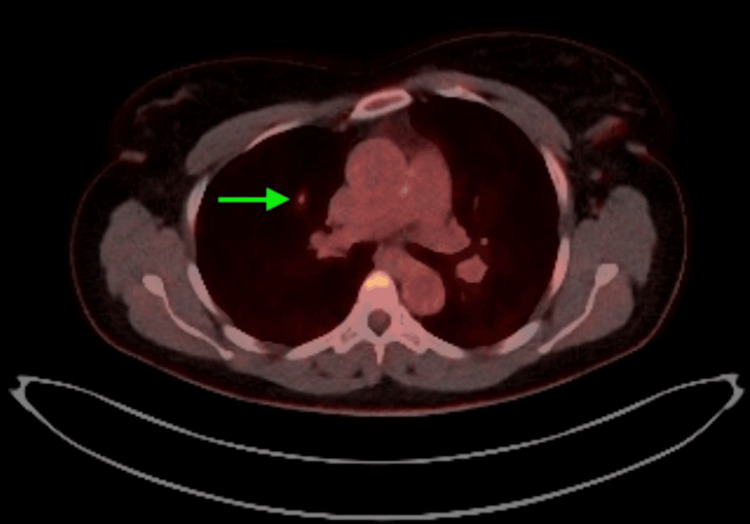
PET-CT scan of a suspicious right upper lobe lesion.

A 45-year-old female was referred for evaluation of a PET-avid right upper lobe pulmonary nodule incidentally discovered during a trauma workup. The lesion, located in the posterior segment of the right upper lobe, measured 9 mm in greatest diameter and exhibited a maximum standardized uptake value (SUVmax) of 6.8 (Figures 1, 2). Despite being peripherally located radiologically, the nodule was not adjacent to the pleura and was suspected to have a partially endobronchial orientation on CT.

Multidisciplinary consensus favored surgical resection. However, given the lesion's size and lack of pleural contact, conventional tactile feedback during VATS was deemed unreliable. Traditional single-coil ICG marking was considered insufficient, as placement distal to the lesion in the segment would not provide reliable three-dimensional localization, and placement proximal to the lesion would not adequately define the segment boundary. Due to this, a decision was made to use a modified bronchoscopic marking technique involving two ICG-soaked coils.

The procedure was performed under superimposed high-frequency jet ventilation (Twinstream; Carl Reiner GmbH, Vienna, Austria) and total intravenous anesthesia.

Flexible bronchoscopy was done using an ultrathin bronchoscope (BF-MP190F; Olympus Medical Systems, Shinjuku, Japan) and a preplanned route to the lesion made using virtual bronchoscopy (Archimedes; Broncus Medical, San Jose, CA, USA) was followed. Radial EBUS (endobronchial ultrasound) was implemented endobronchially to confirm the lesion's location.

**Figure 3:**
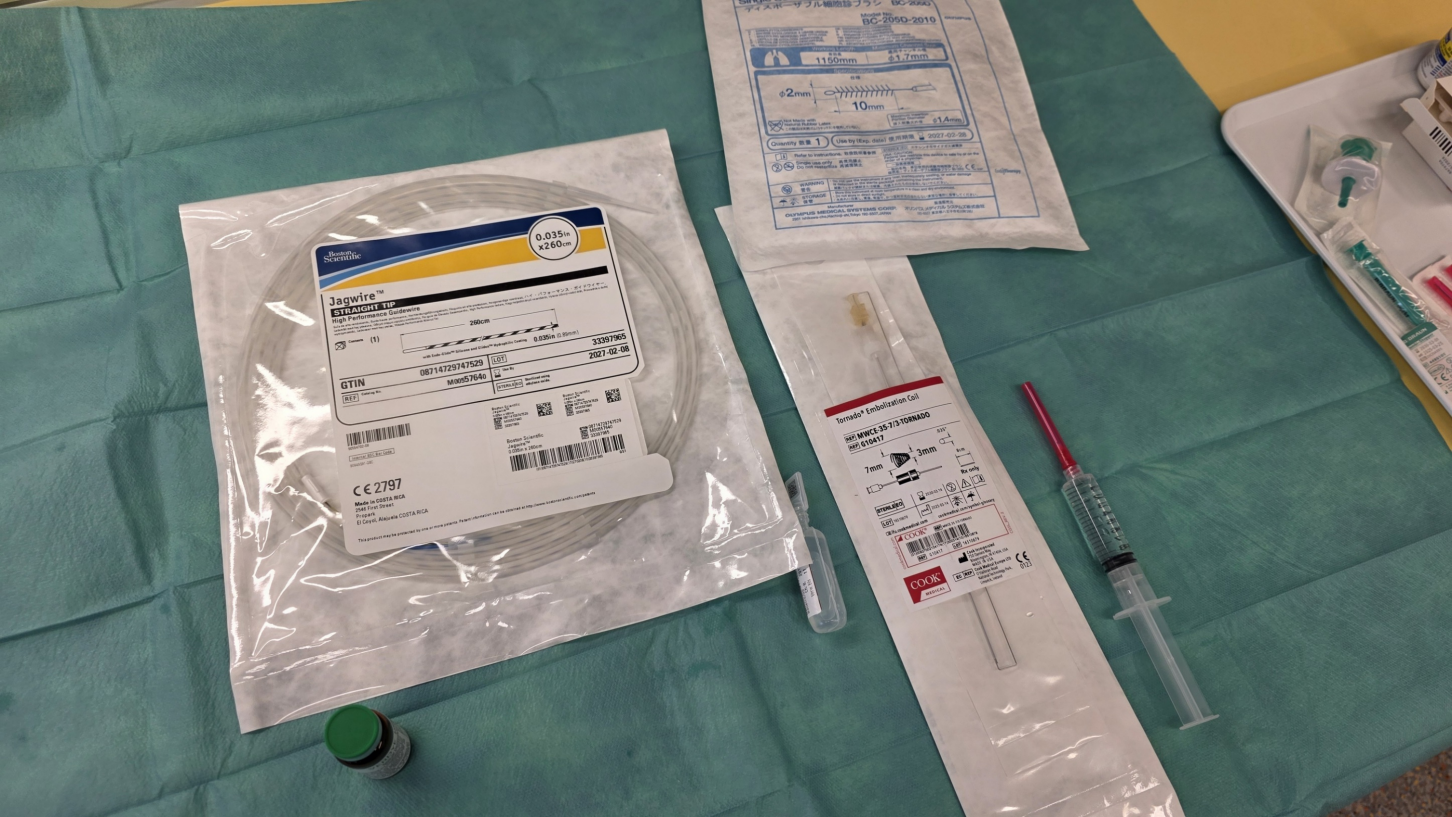
Materials: ICG dye, guide wire, cytology brush, and embolization coil. ICG: indocyanine green

Two embolization coils (MWCE-35-6/3 Tornado embolization coil, 6 × 3 mm, and MWCE-35-7/3 Tornado embolization coil, 7 × 3 mm; Cook Medical, Baesweiler, Germany) were soaked with ICG dye (VERDYE; Diagnostic Green, Farmington Hills, MI, USA) for 10 minutes before placement (Figure 3).

The soaked coils were then retrogradely loaded into a 1.7 mm cytology brush's catheter (BC-205D-2010; Olympus Medical Systems) with the aid of a guide wire to push the coils out of their cartridges.

**Figure 4:**
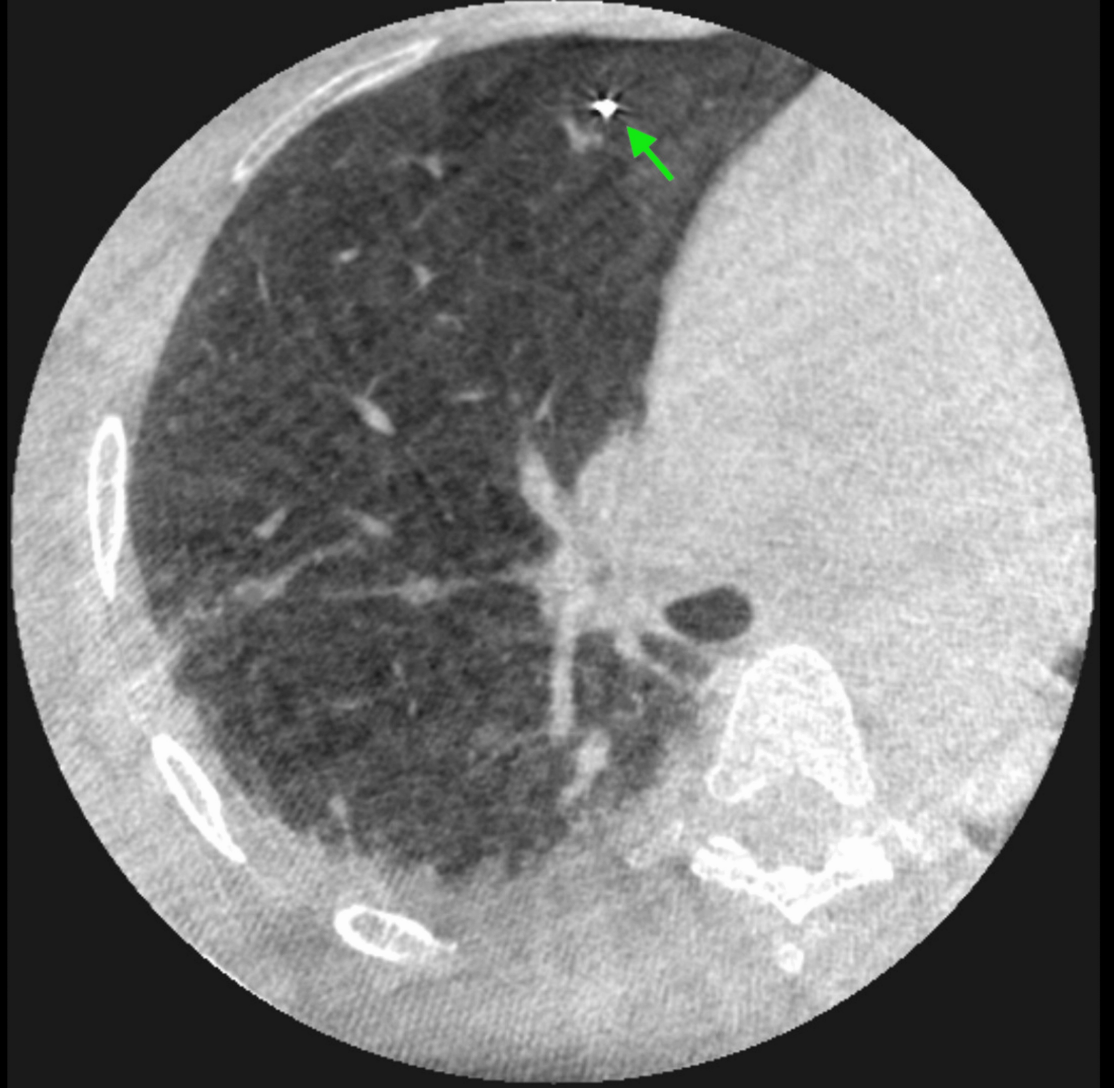
CBCT image of brush catheter adjacent to pleura (axial view). CBCT: cone-beam computed tomography

**Figure 5:**
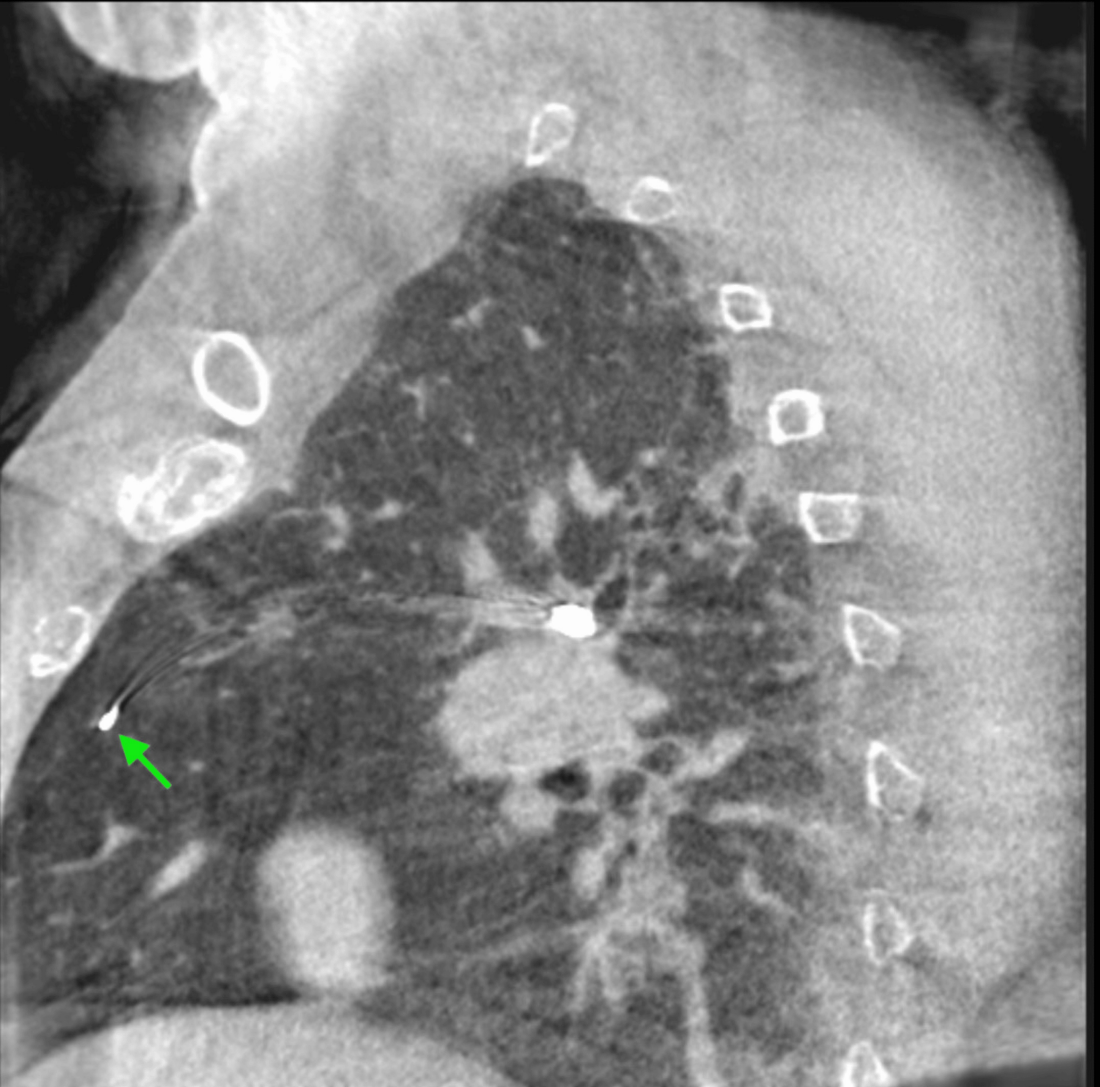
CBCT image of brush catheter adjacent to pleura (sagittal view). CBCT: cone-beam computed tomography

**Figure 6:**
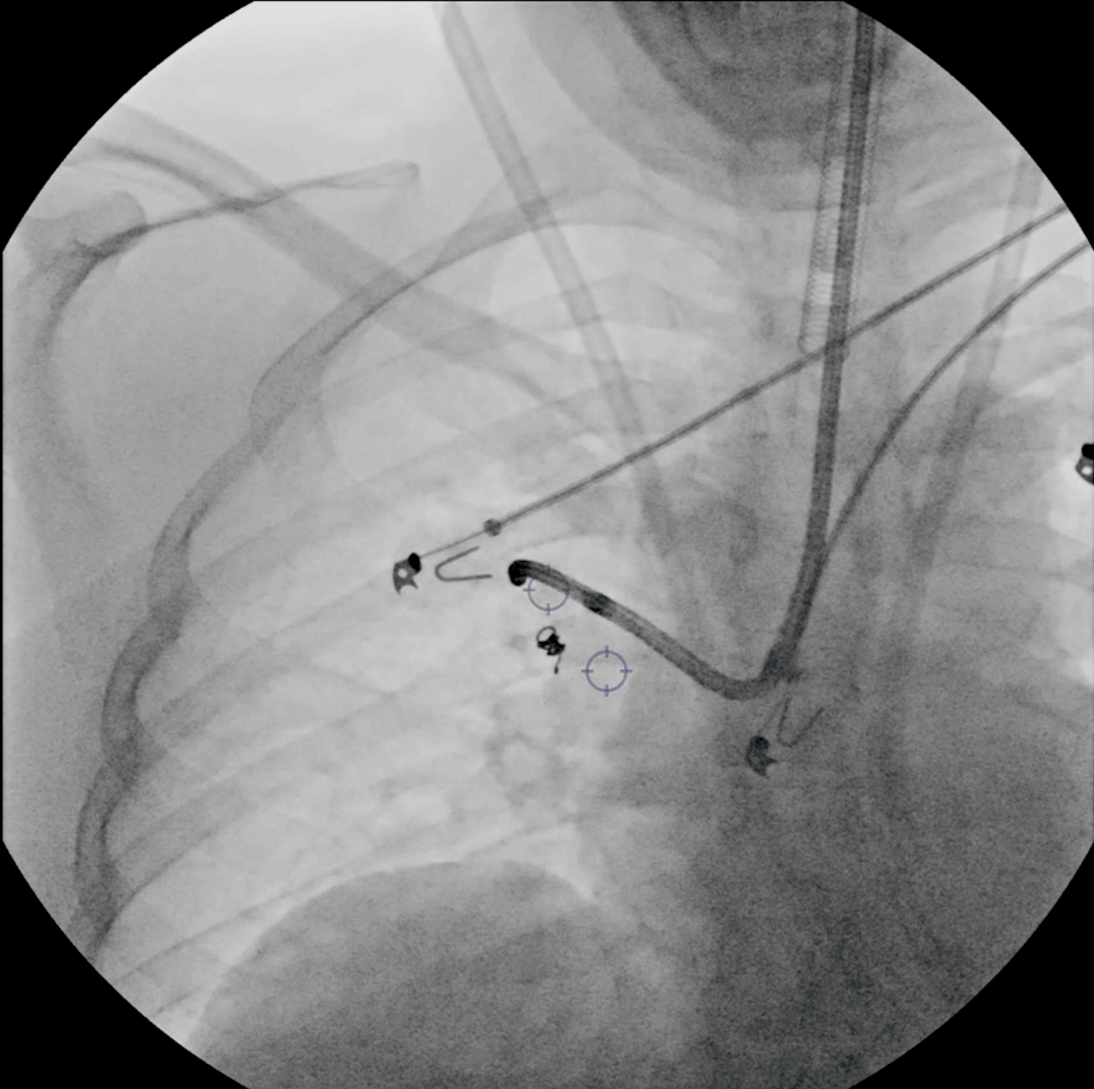
Deployment of the ICG-soaked coil under augmented fluoroscopy. ICG: indocyanine green

The first ICG-soaked coil was bronchoscopically deployed distally within the posterior segment, beyond the lesion and adjacent to the pleura, using cone-beam computed tomography (CBCT) guidance (Figures 4-6). Due to the angulated position of the bronchoscope and the brush catheter's close proximity to the pleura, the cytology brush was not used for deployment, as significant resistance was encountered. Instead, the brush was removed from the catheter, and the guidewire was advanced into the catheter to carefully push the coil into place. This method enabled an atraumatic deployment and minimized the risk of pneumothorax. This first coil served to provide the surgeon with a fluorescent marker to delineate the distal segmental boundary relative to the lesion.

**Figure 7:**
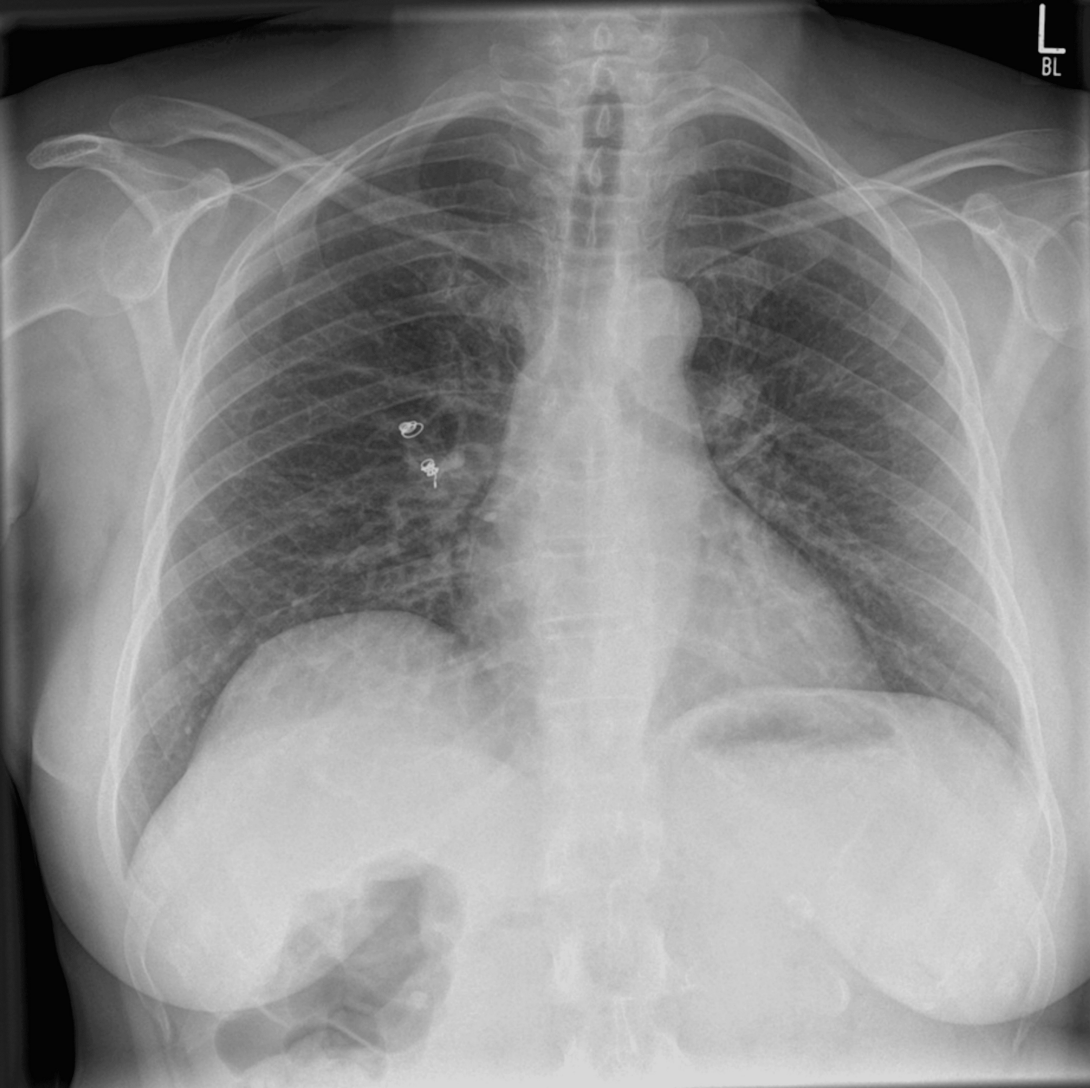
Postinterventional chest X-ray showing coil placement.

The second ICG-soaked coil was deployed endobronchially, just proximal and adjacent to the lesion, following precise CBCT navigation alignment and using the cytology brush as a deployment tool. This second coil served as a direct marker for the tumor location. The spatial separation between the distal and proximal coils measured approximately 37 mm on CBCT imaging, ensuring a reliable three-dimensional bracket of the lesion. This spacing avoided overlapping fluorescence signals while still providing precise orientation for resection. The bronchoscopy, including coil deployment, required approximately 55 minutes. A post-intervention chest X-ray confirmed positioning of both coils and absence of pneumothorax (Figure 7).

**Figure 8:**
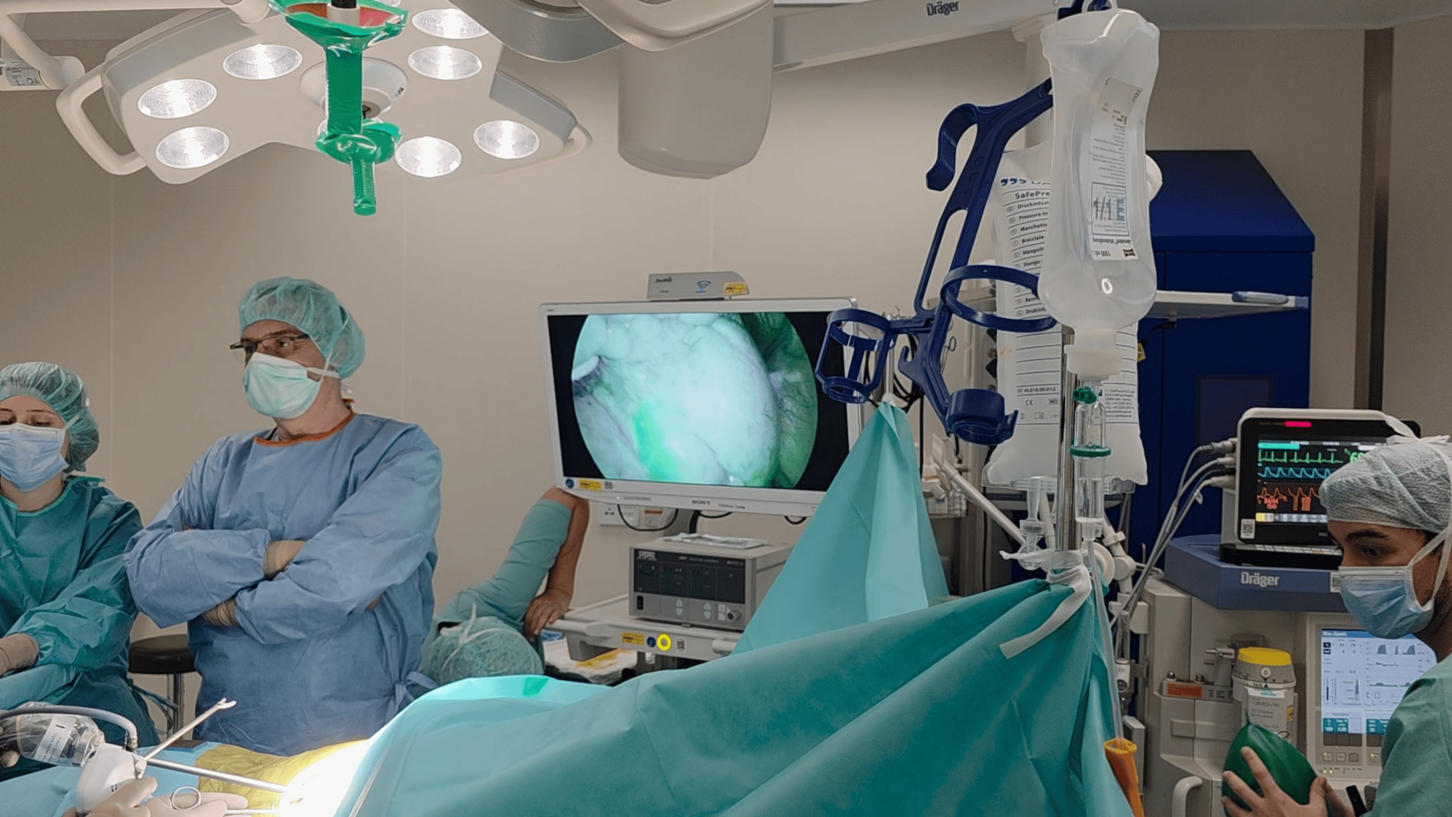
Localization of the first coil (adjacent to the pleura) using infrared imaging during uniportal VATS. VATS: video-assisted thoracoscopic surgery Original image by the authors.

**Figure 9:**
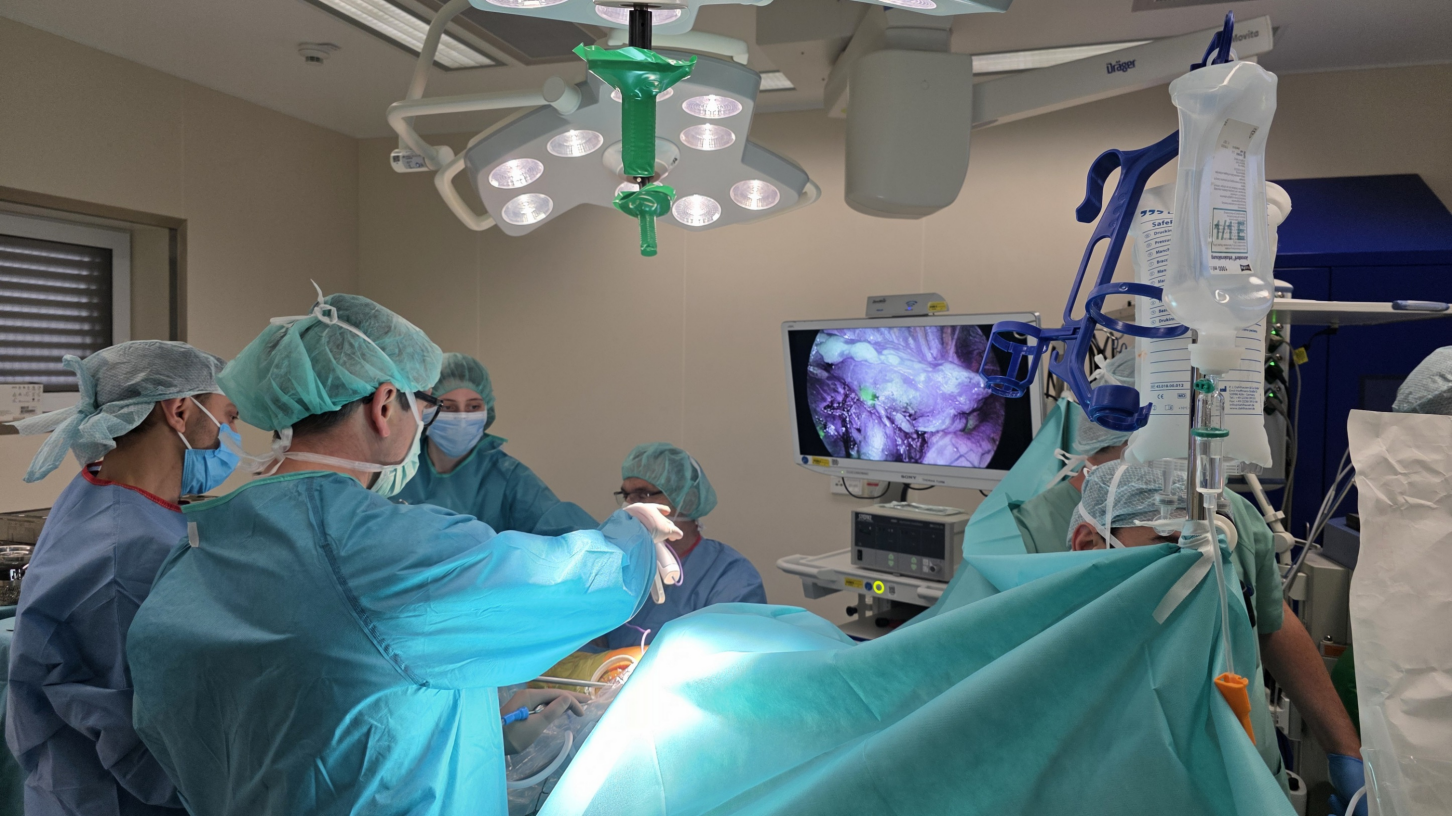
Localization of the second coil (proximal to the tumor) using infrared imaging during uniportal VATS. VATS: video-assisted thoracoscopic surgery Original image by the authors.

The patient underwent surgery three days after implantation of the coils. The lesion was successfully resected via uniportal VATS without the need for conversion to thoracotomy. Both coils were visualized clearly under near-infrared fluorescence during VATS (Figures 8, 9).

**Figure 10:**
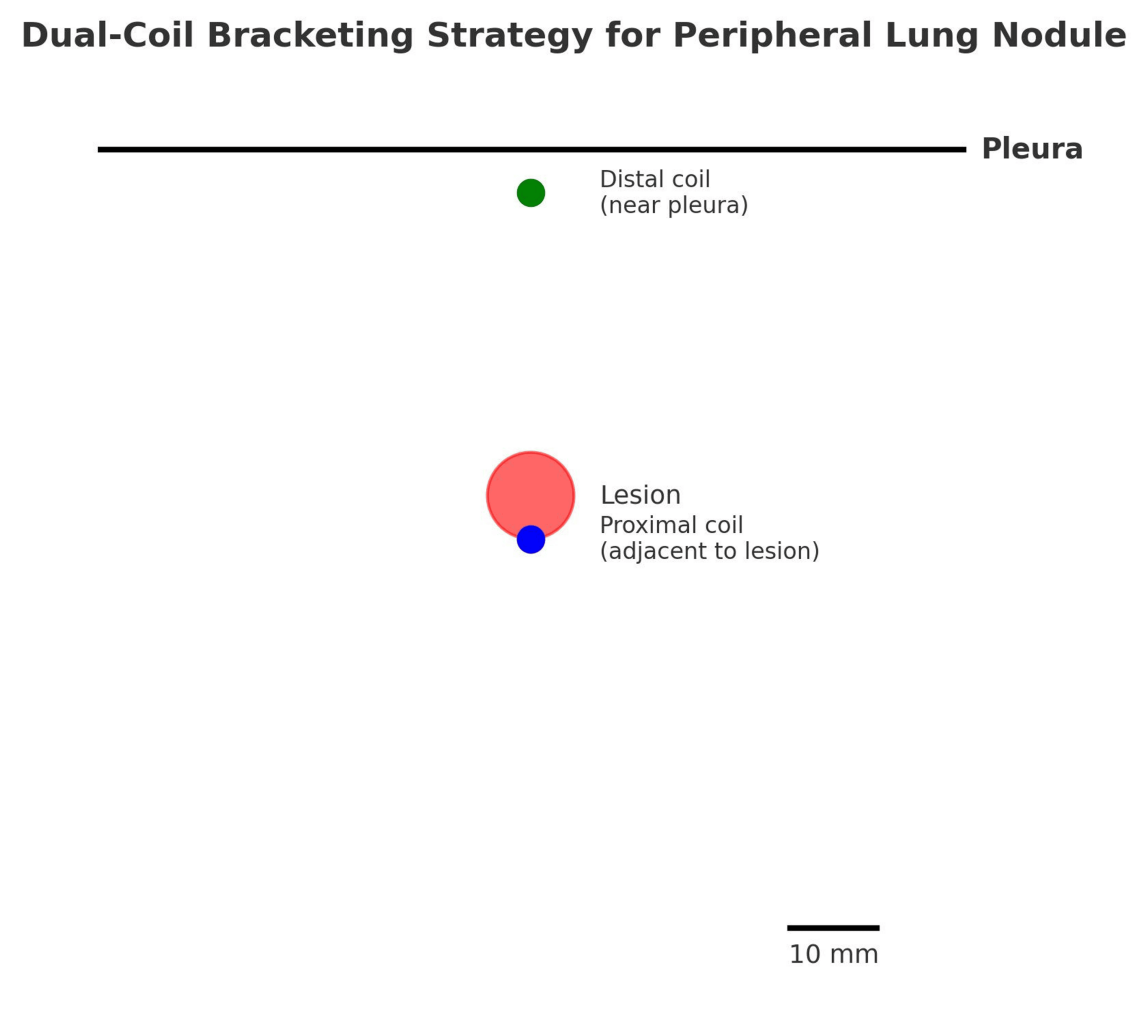
Schematic of dual-coil bracketing Image Credit: Sammy Onyancha

**Figure 11:**
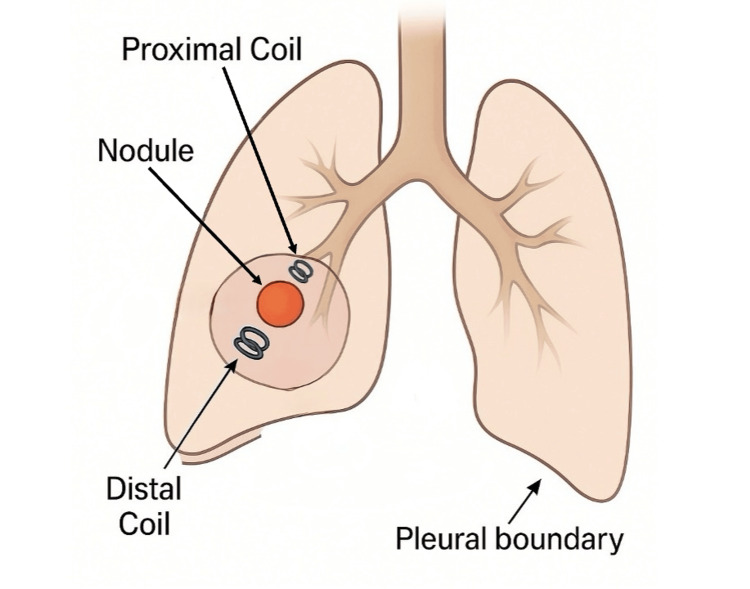
Nodule bracketing with dual coils Image Credit: Sammy Onyancha

The dual-marker approach provided a spatial "bracketing" of the tumor, enabling the surgeon to confidently triangulate the lesion's position (Figures 10, 11) (Video 1). The uniportal VATS resection lasted 118 minutes, consistent with operative times for single-coil localization procedures. No delays or intraoperative challenges attributable to localization were encountered. Final pathology confirmed a typical carcinoid with clear margins. The patient had an uneventful postoperative course and was discharged on the fourth postoperative day.

**Video 1:** Step-by-step guide: dual ICG-soaked coil placement ICG: indocyanine green

## Discussion

This case highlights a novel adaptation of the conventional ICG-soaked coil localization technique. While single-coil techniques are effective for subpleural nodules, they are inadequate when the tumor is situated deeper within the parenchyma.

Our modified approach of using two coils, one distal to mark the segment boundary and another proximal to the lesion, facilitates precise localization in three dimensions and eliminates the need for tactile localization. It may be particularly advantageous for small (≤15 mm), PET-avid nodules that are radiologically peripheral but not in direct contact with the pleura, where tactile localization during VATS is unreliable. It may also be useful when lesions demonstrate partial endobronchial orientation or are located at a segmental level where single-coil placement cannot provide sufficient three-dimensional guidance.

By clearly demarcating both the lesion and the relevant segmental anatomy, the technique supports precise anatomical resections, reducing the risk of excessive parenchymal removal. This is particularly important in younger patients or those with limited pulmonary reserve, where preserving functional lung tissue has long-term benefits.

Moreover, the integration of this localization strategy with minimally invasive surgery, specifically uniportal VATS, offers significant advantages, as it is associated with reduced postoperative pain, fewer complications, better cosmetic outcomes, and faster recovery compared to multiport VATS or thoracotomy [17-19]. In our case, the precise localization allowed for a confident, targeted resection via uniportal VATS, thereby avoiding the need for conversion to thoracotomy. The patient had an uneventful postoperative course and was discharged on postoperative day four, reflecting the efficiency and patient-centered value of this approach.

Additionally, the entire procedure was performed using conventional flexible bronchoscopy, without the need for robotic assistance, which highlights the accessibility of the method.

Although promising, the dual-coil technique requires an additional embolization coil and slightly longer bronchoscopy time compared to single-coil methods (42 minutes vs. 55 minutes). In our setting, the incremental material cost is approximately €85, which is modest when weighed against the clinical benefits of preventing conversion to thoracotomy or avoiding excessive lung resection. Institutions considering adoption may find this approach cost-effective, particularly in anatomically challenging cases.

Qualitatively, the intraoperative fluorescence was graded by the surgical team as highly distinct, with both coils clearly visible under near-infrared imaging. Although no tool for measuring quantitative fluorescence intensity was used in this case, future studies should consider semi-quantitative visibility scores or photometric intensity assessments. Such objective metrics would allow reproducible comparisons across different patients and localization strategies.

Furthermore, several potential complications have to be considered. Coil migration remains a theoretical risk, though this can be minimized by selecting appropriately sized coils and confirming placement under CBCT guidance. Pneumothorax or parenchymal trauma may occur if the catheter is advanced forcefully against resistance, highlighting the importance of atraumatic deployment as demonstrated in our case by using a guidewire instead of the cytology brush when high resistance was encountered. Another potential challenge is the loss or attenuation of fluorescence signals, particularly in obese patients or when nodules are located deeper within the parenchyma. In such scenarios, dual-coil bracketing offers redundancy; even if one marker is suboptimally visualized, the second coil provides spatial orientation and ensures reliable intraoperative localization. Together, these technical safeguards enhance the reproducibility and safety of the dual-coil method while reducing the likelihood of conversion to open thoracotomy. However, technical feasibility depends on the bronchoscopic accessibility of the target segment.

## Conclusions

This dual-coil ICG localization strategy expands the utility of fluorescence-guided surgery to include non-subpleural and centrally oriented nodules. It facilitates lung-sparing, minimally invasive resections with high precision while avoiding thoracotomy, which is particularly advantageous in small, impalpable lesions. Further studies in larger cohorts are required to validate the technique and to determine its reproducibility, optimal indications, and long-term outcomes compared to conventional localization methods.

## References

[1] Fiorelli A, Forte S, Santini M, Petersen RH, Fang W (2022). Did conversion to thoracotomy during thoracoscopic lobectomy increase post-operative complications and prejudice survival? Results of best evidence topic analysis. Thorac Cancer.

[2] Suzuki K, Nagai K, Yoshida J, Ohmatsu H, Takahashi K, Nishimura M, Nishiwaki Y (1999). Video-assisted thoracoscopic surgery for small indeterminate pulmonary nodules: indications for preoperative marking. Chest.

[3] Imperatori A, Nardecchia E, Cattoni M (2021). Perioperative identifications of non-palpable pulmonary nodules: a narrative review. J Thorac Dis.

[4] Sun X, Fu J, Ma C, Song Z, Yang S, Jin L, Duan F (2024). CT-guided microcoil versus hook-wire localization of pulmonary nodule prior to video-assisted thoracoscopic surgery without fluoroscopic guidance. BMC Pulm Med.

[5] Park CH, Han K, Hur J (2017). Comparative effectiveness and safety of preoperative lung localization for pulmonary nodules: a systematic review and meta-analysis. Chest.

[6] Song JW, Park IK, Bae SY, Na KJ, Park S, Kang CH, Kim YT (2022). Electromagnetic navigation bronchoscopy-guided dye marking for localization of pulmonary nodules. Ann Thorac Surg.

[7] Folch EE, Bowling MR, Pritchett MA (2022). NAVIGATE 24-month results: electromagnetic navigation bronchoscopy for pulmonary lesions at 37 centers in Europe and the United States. J Thorac Oncol.

[8] Anayama T, Hirohashi K, Miyazaki R, Okada H, Yamamoto M, Orihashi K (2021). Fluorescence visualization of the intersegmental plane by bronchoscopic instillation of indocyanine green into the targeted segmental bronchus: determination of the optimal settings. J Int Med Res.

[9] Xu Y, Liu H, Qin Y, Guo C, Li S, Liang N (2024). Localization strategies for deep lung nodule using electromagnetic navigation bronchoscopy and indocyanine green fluorescence: a technical note. J Thorac Dis.

[10] Bowling MR, Folch EE, Khandhar SJ (2019). Pleural dye marking of lung nodules by electromagnetic navigation bronchoscopy. Clin Respir J.

[11] Bawaadam H, Benn BS, Colwell EM, Oka T, Krishna G (2023). Lung nodule marking with ICG dye-soaked coil facilitates localization and delayed surgical resection. Ann Thorac Surg Short Rep.

[12] Chan JW, Chang AT, Yu PS, Lau RW, Ng CS (2022). Robotic assisted-bronchoscopy with cone-beam CT ICG dye marking for lung nodule localization: experience beyond USA. Front Surg.

[13] Sincavage J, Gulack BC, Zamora IJ (2024). Indocyanine green (ICG) fluorescence-enhanced applications in pediatric surgery. Semin Pediatr Surg.

[14] Libor L, Pécsy B, Szűcs E, Lantos J, Bakos A, Lázár G, Furák J (2024). Effect of transbronchial or intravenous administration of indocyanine green on resection margins during near-infrared-guided segmentectomy: a review. Front Surg.

[15] Okusanya OT, Hess NR, Luketich JD, Sarkaria IS (2018). Infrared intraoperative fluorescence imaging using indocyanine green in thoracic surgery. Eur J Cardiothorac Surg.

[16] Chiba R, Ebihara Y, Shiiya H (2022). A novel system for analyzing indocyanine green (ICG) fluorescence spectra enables deeper lung tumor localization during thoracoscopic surgery. J Thorac Dis.

[17] Nachira D, Congedo MT, Tabacco D (2022). Surgical effectiveness of uniportal-VATS lobectomy compared to open surgery in early-stage lung cancer. Front Surg.

[18] Li T, Xia L, Wang J, Xu S, Sun X, Xu M, Xie M (2021). Uniportal versus three-port video-assisted thoracoscopic surgery for non-small cell lung cancer: a retrospective study. Thorac Cancer.

[19] Harris CG, James RS, Tian DH, Yan TD, Doyle MP, Gonzalez-Rivas D, Cao C (2016). Systematic review and meta-analysis of uniportal versus multiportal video-assisted thoracoscopic lobectomy for lung cancer. Ann Cardiothorac Surg.

